# Risk of subsequent ventricular arrhythmia is higher in primary prevention patients with implantable cardioverter defibrillator than in secondary prevention patients

**DOI:** 10.1186/s12872-019-1218-9

**Published:** 2019-10-21

**Authors:** You Zhou, Shuang Zhao, Keping Chen, Wei Hua, Yangang Su, Silin Chen, Zhaoguang Liang, Wei Xu, Shu Zhang

**Affiliations:** 1 State Key Laboratory of Cardiovascular Disease, Arrhythmia Center, Fuwai Hospital, National Center for Cardiovascular Diseases, Chinese Academy of Medical Sciences and Peking Union Medical College, 167 Bei Li Shi Road, Xicheng District, Beijing, 100037 China; 2 Department of Cardiology, Shanghai Institute of Cardiovascular Diseases, Zhongshan Hospital, Fudan University, Shanghai, China; 3 Department of Cardiology, Guangdong Cardiovascular Institute, Guangdong General Hospital, Guangzhou, China; 4 Department of Cardiology, First Affiliated Hospital of Harbin Medical University, Harbin, China; 5 Department of Cardiology, Nanjing Drum Tower Hospital, Nanjing, China

**Keywords:** Implantable cardioverter defibrillator, Ventricular arrhythmia, Primary prevention, Secondary prevention, Home monitoring

## Abstract

**Background:**

Because of previous ventricular arrhythmia (VA) episodes, patients with implantable cardioverter-defibrillator (ICD) for secondary prevention (SP) are generally considered to have a higher burden of VAs than primary prevention (PP) patients. However, when PP patients experienced VA, the difference in the prognosis of these two patient groups was unknown.

**Methods:**

The clinical characteristics and follow-up data of 835 ICD patients (364 SP patients and 471 PP patients) with home monitoring feature were retrospectively analysed. The incidence rate and risk of subsequent VA and all-cause mortality were compared between PP patients after the first appropriate ICD therapy and SP patients.

**Results:**

During a mean follow-up of 44.72 ± 20.87 months, 210 (44.59%) PP patients underwent appropriate ICD therapy. In the Kaplan-Meier survival analysis, the PP patients after appropriate ICD therapy were more prone to VA recurrence and all-cause mortality than SP patients (*P*<0.001 for both endpoints). The rate of appropriate ICD therapy and all-cause mortality in PP patients after the first appropriate ICD therapy was significantly higher than that in SP patients (for device therapy, 59.46 vs 20.64 patients per 100 patient-years; incidence rate ratio [IRR] 2.880, 95% confidence interval [CI]: 2.305–3.599; *P*<0.001; for all-cause mortality, 14.08 vs 5.40 deaths per 100 patient-years; IRR 2.607, 95% CI: 1.884–3.606; *P*<0.001). After propensity score matching for baseline characteristics, the risk of VA recurrence in PP patients with appropriate ICD therapy was still higher than that in SP patients (41.80 vs 19.10 patients per 100 patient-years; IRR 2.491, 95% CI: 1.889–3.287; *P*<0.001), but all-cause mortality rates were similar between the two groups (12.61 vs 9.33 deaths per 100 patient-years; IRR 1.352, 95% CI: 0.927–1.972; *P* = 0.117).

**Conclusions:**

Once PP patients undergo appropriate ICD therapy, they will be more prone to VA recurrence and death than SP patients.

## Background

The implantable cardioverter-defibrillator (ICD) has been proven by multiple trials to be efficacious in identifying and terminating malignant ventricular arrhythmias (VAs) to prevent sudden cardiac death (SCD) since late 1990s [1–4]. Initially, patients were treated with ICD implantation after survival of a life-threatening VA (secondary prevention, SP), but because of the low survival rate after experiencing VA, focus shifted to the identification of patients at high risk of SCD (primary prevention, PP). Despite the survival benefit revealed in clinical trials [3, 4], ICDs are not a panacea suitable for every patient with left ventricular dysfunction. Although the role of ICDs in SP is well-established, identification of the appropriate candidate who will benefit from PP implantation remains a challenge. Because of previous VA episodes, SP patients are generally considered to have a higher burden of VAs than PP patients [5]. However, many PP patients never experienced VA requiring ICD therapy before devices’ battery depletion or death.

When PP patients experienced VA, becoming a “survivor” similar to SP patients, the difference in prognosis of these two population was unknown. Due to the prophylactic use of ICD, many PP patients who would have died because of malignant VA survived. Those patients may have the highest risk of SCD, even worse than SP patients. To evaluate the prognosis of those patients will help us identify high SCD risk patients and improve patient management. Thus, this study aimed to compare the incidence of subsequent VA episodes and all-cause mortality between PP patients with appropriate ICD therapy and SP patients in an ICD registry study from China.

## Methods

The present study was a retrospective analysis of archived HM transmission data from Biotronik SUMMIT registry study in China [Study of Home Monitoring System Safety and Efficacy in Cardiac Implantable Electronic Device implanted patients (SUMMIT)]. The protocol of SUMMIT study followed the Declaration of Helsinki and was approved by the Hospital Ethics Committee. All patients provided written informed consent before SUMMIT study participation. All ICD patients with complete daily HM data in SUMMIT study were enrolled in the present analysis.

Device programming were as follows. The basic pacing rate was 40–60 bpm. Tachycardia detection and therapy programming included 3 zones: ventricular tachycardia (VT) monitor zone (140–170 bpm), VT therapy zone (170–210 bpm, 2–3 bursts of ATP followed by high-energy shock if episodes persisting), and ventricular fibrillation (VF) zone (> 210 bpm, high-energy shock alone). The detection interval in VT therapy zone was 26 beats with a 20-beat redetection, and 12 of 16 beats in VF zone. The discrimination algorithm was Biotronik SMART® algorithm. HM were programmed on to provide continuous data transmission. Other programmable parameters were at the discretion of individual physicians.

The primary endpoint was the appropriate ICD therapy of VT/VF, and the secondary endpoint was all-cause mortality. ICD therapies were confirmed by intracardiac electrograms from stored HM data. Inappropriate events were excluded from the analysis. Once the patient’s HM transmission was disrupted, the status of the patient was confirmed by telephone. If the patient was confirmed to have died, the date and cause of death were acquired by contacting the family.

We compared the incidence rate and risk of subsequent VA and all-cause mortality between PP patients after the first appropriate ICD therapy and SP patients. The SP patients had experienced VA before device implantation, but PP patients had not. Therefore, the risk of a first appropriate ICD therapy in SP patients was compared with the risk of the second appropriate ICD therapy in PP patients.

Propensity score matching was performed considering the differences in baseline characteristics between patients of different indications. The matching was estimated by a multivariable logistic regression model. The covariates included in the model were age, sex, left ventricular ejection fraction (LVEF), left ventricular end-diastolic diameter, New York Heart Association class, renin–angiotensin system blockers, diuretics, and amiodarone. The propensity score matching was performed with a ratio of 1:1 and a calliper of 0.1.

Baseline clinical characteristics were presented using medians (±SDs) for continuous variables and percentages for categorical variables. Group comparisons were performed using chi-square tests for categorical variables and Student t tests or Mann-Whitney U tests for continuous variables. Kaplan-Meier methods were used to create survival curves, and the log-rank test was used for comparison. The rates of appropriate ICD therapy and death were computed for 100 patient-years and compared using the means of Poisson regression to report incidence rate ratio (IRR). A *P* value < 0.05 was considered statistically significant. STATA 14 (StataCorp LLC, College Station, TX, USA) and GraphPad Prism 6 (GraphPad Software, Inc., La Jolla, CA, USA) were used to perform the statistical analysis.

## Results

Nine hundred ten patients with ICD or CRT-D were included in SUMMIT registry. 75 (8.2%) patients were not included in analysis for no HM data transmission. Eight hundred thirty-five patients were included in this study, consisting of 364 (43.59%) SP patients and 471 (56.41%) PP patients. During the mean follow-up duration of 44.72 ± 20.87 months, 210 (44.59%) PP patients underwent appropriate ICD therapy (ATP or shock). Compared with PP patients who never underwent appropriate ICD therapy, more PP patients who had undergone appropriate ICD therapy had ischemic heart disease (Table 1).

**Table 1:** Baseline characteristics of primary prevention patients experienced VA vs. no VA

Baseline characteristics	Patients experienced VA(*n* = 210)	Patients not experienced VA (*n* = 261)	*P*-value
Male	166 (79.0%)	193 (73.9%)	0.231
Age(years)	62.72 ± 11.83	61.50 ± 13.77	0.475
Ischemic heart disease	57 (27.1%)	48 (18.4%)	0.023
Hypertension	70 (33.3%)	97 (37.2%)	0.438
Diabetes	28 (13.3%)	27 (10.3%)	0.317
Stroke	4 (1.9%)	6 (2.3%)	1.000
Atrial fibrillation	26 (12.4%)	18 (6.9%)	0.055
LVEF (%)	33.13 ± 10.64	34.29 ± 9.48	0.218
LVEDD (mm)	64.68 ± 12.94	63.48 ± 11.40	0.575
NYHA class III-IV	129 (61.4%)	169 (64.8%)	0.457
Beta-blockers	129 (61.4%)	145 (55.6%)	0.222
ACEI/ARB	88 (43.8%)	113 (43.3%)	0.852
Diuretics	77 (36.7%)	102 (39.1%)	0.633
Amiodarone	47 (22.4%)	47 (18.0%)	0.248

*Abbreviations*: *VA* Ventricular arrhythmia, *LVEF* Left ventricular Ejection fraction, *LVEDD* Left ventricular end-diastolic diameter, *NYHA class* New York Heart Association class, *ACEI* Angiotensin-converting enzyme inhibitor, *ARB* Angiotensin receptor blocker

Table 2 showed that PP patients who experienced VA were older, were men, and had higher New York Heart Association functional class, lower LVEF, and larger left ventricular end-diastolic diameter; these patients were more likely to be treated with renin-angiotensin system blockers, spironolactone, and diuretics and less likely to be treated with amiodarone than SP patients.

**Table 2:** Baseline characteristics of primary prevention patients experienced VA vs. secondary prevention patients

Baseline characteristics	Before matching			After matching		
	Primary Prevention experienced VA(*n* = 210)	Secondary Prevention (*n* = 364)	*P*-value	Primary Prevention experienced VA(*n* = 173)	Secondary Prevention (*n* = 173)	*P*-value
Male	166 (79.0%)	259 (71.2%)	0.038	135 (78.0%)	126 (72.8%)	0.318
Age(years)	62.72 ± 11.83	58.30 ± 13.93	0.001	62.58 ± 11.77	64.00 ± 12.80	0.115
Ischemic heart disease	57 (27.1%)	111 (30.5%)	0.446	47 (27.2%)	61 (35.3%)	0.131
Hypertension	70 (33.3%)	104 (28.6%)	0.258	53 (30.6%)	54 (31.2%)	0.907
Diabetes	28 (13.3%)	34 (9.3%)	0.162	21 (12.1%)	21 (12.1%)	1.000
Stroke	4 (1.9%)	7 (1.9%)	1.000	3 (1.7%)	4 (2.3%)	1.000
Atrial fibrillation	26 (12.4%)	33 (9.1%)	0.253	24 (13.9%)	21 (12.1%)	0.632
LVEF (%)	33.13 ± 10.64	43.18 ± 12.00	<0.001	34.73 ± 10.83	36.33 ± 11.00	0.116
LVEDD (mm)	64.68 ± 12.94	57.83 ± 10.74	<0.001	63.84 ± 13.25	62.87 ± 10.80	0.707
NYHA class III-IV	129 (61.4%)	117 (32.1%)	<0.001	97 (56.1%)	95 (54.9%)	0.829
Beta-blockers	129 (61.4%)	210 (57.7%)	0.428	107 (61.8%)	99 (57.2%)	0.381
ACEI/ARB	88 (43.8%)	92 (24.2%)	<0.001	68 (39.3%)	60 (34.7%)	0.373
Diuretics	77 (36.7%)	69 (19.0%)	<0.001	56 (32.4%)	48 (27.7%)	0.348
Amiodarone	47 (22.4%)	129 (35.4%)	0.001	45 (26.0%)	52 (30.1%)	0.404

*Abbreviations*: *VA* Ventricular arrhythmia, *LVEF* Left ventricular Ejection fraction, *LVEDD* Left ventricular end-diastolic diameter, *NYHA class* New York Heart Association class, *ACEI* Angiotensin-converting enzyme inhibitor, *ARB* Angiotensin receptor blocker

**Fig. 1:**
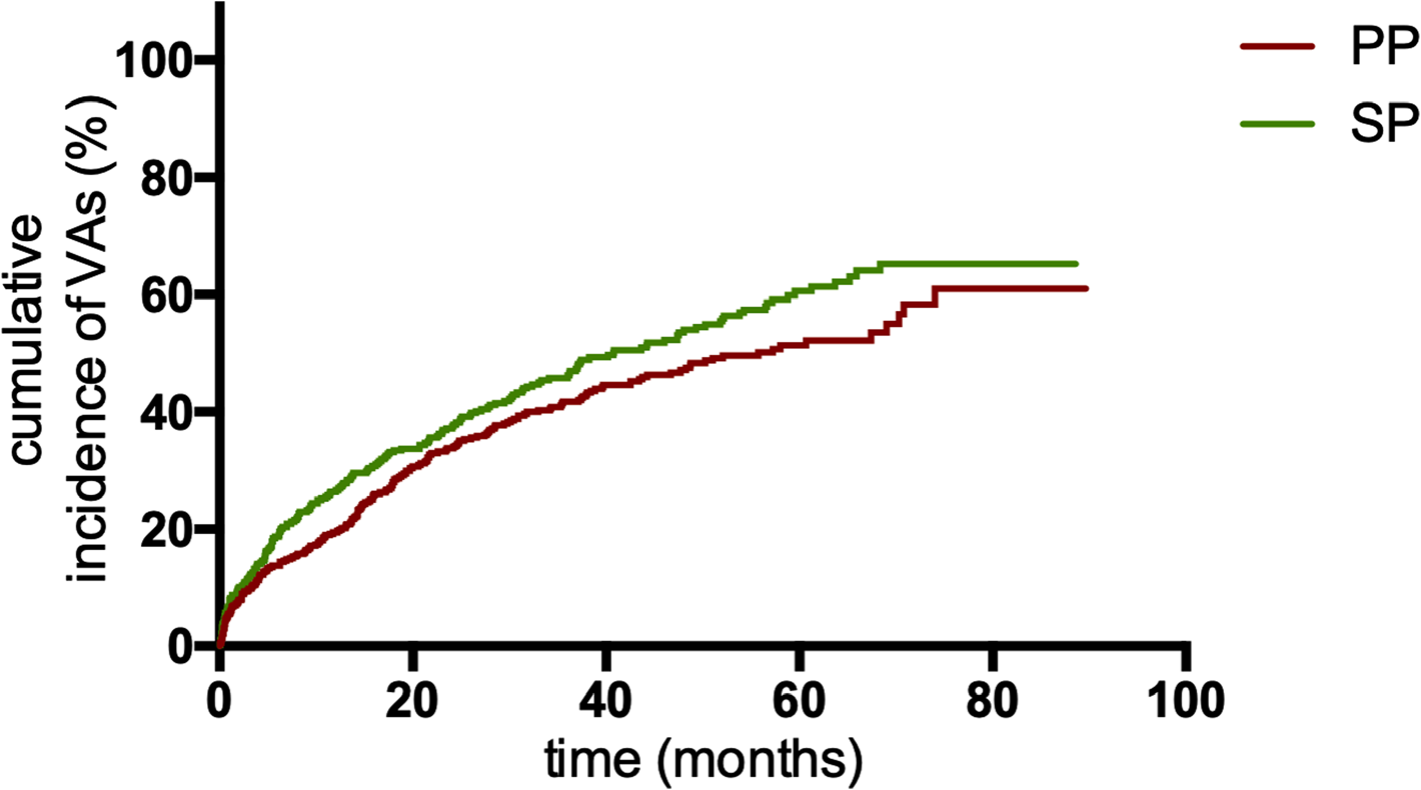
Kaplan-Meier estimates of the cumulative incidence of VAs between PP patients and SP patients. Abbreviations: VA, ventricular arrhythmia; PP, primary prevention; SP, secondary prevention

**Fig. 2:**
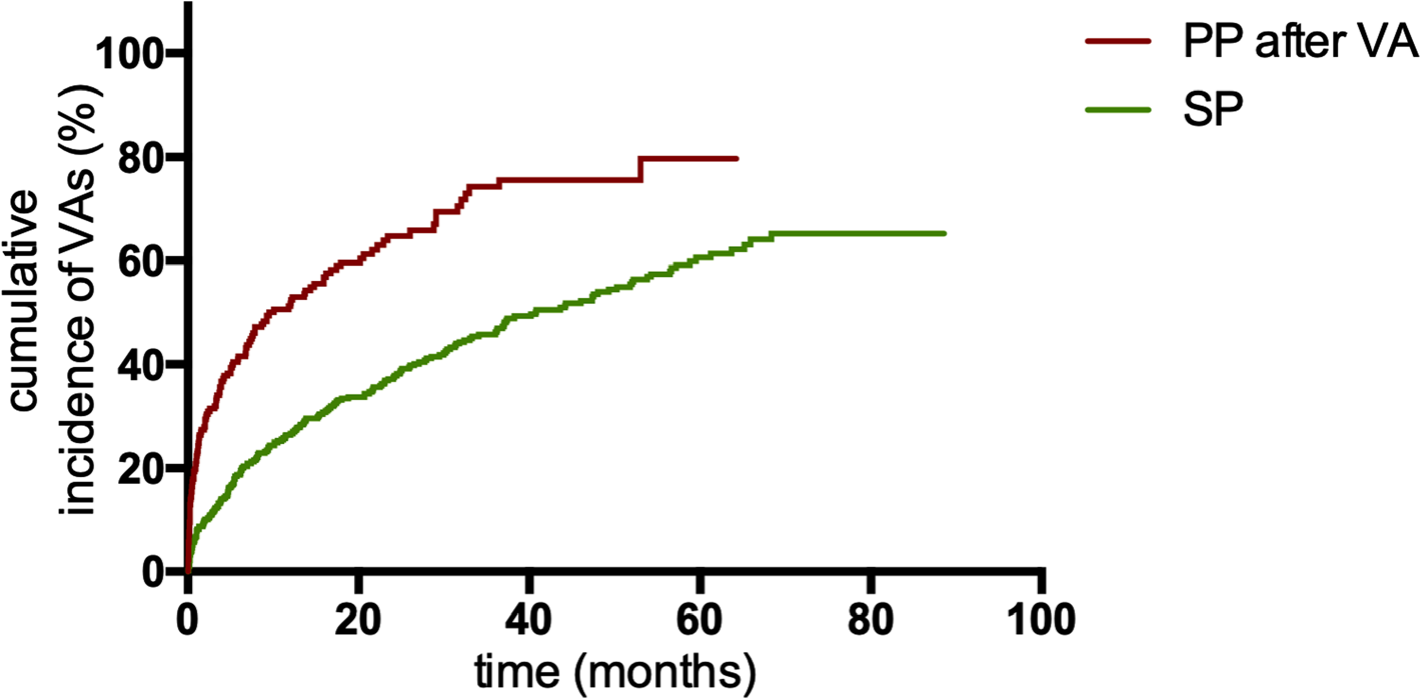
Kaplan-Meier estimates of the cumulative incidence of VAs between PP patients after appropriate device therapies and SP patients. Abbreviations: VA, ventricular arrhythmia; PP, primary prevention; SP, secondary prevention

VA triggered appropriate ICD therapy in 193 (53.02%) SP patients. The Kaplan-Meier analysis of time from device implantation to first appropriate therapy showed a significant higher incidence of appropriate ICD therapies among SP patients than PP patients (*P* = 0.044; Fig. 1). Of 210 PP patients with first appropriate ICD therapy, 129 (61.43%) underwent a second appropriate device therapy. Comparison of these groups demonstrated that PP ICD recipients had a higher risk of a subsequent appropriate therapy recurrence after the first therapy than SP patients with the first appropriate therapy (*P*<0.001; Fig. 2). In addition, the rate of appropriate therapies in PP patients after the first appropriate ICD therapy was also significantly higher than that in SP patients (59.46 vs 20.64 patients per 100 patient-years; IRR 2.880, 95% confidence interval [CI]: 2.305–3.599; *P*<0.001; Table 3).

**Table 3:** Rate of endpoints according to indications

	Primary Prevention patients with VA	Secondary Prevention	Incidence rate ratio^a^	*P*-Value
Before matching				
Rate of Appropriate therapy (per 100 patients-year)	59.46 (55.30–63.62)	20.64 (18.33–22.96)	2.880 (2.305–3.599)	<0.001
Rate of All-cause mortality (per 100 patients-year)	14.08 (11.27–16.90)	5.40 (4.25–6.56)	2.607 (1.884–3.606)	<0.001
After matching				
Rate of Appropriate therapy (per 100 patients-year)	41.80 (37.19–46.41)	19.10 (15.98–22.22)	2.491 (1.889–3.287)	<0.001
Rate of All-cause mortality (per 100 patients-year)	12.61 (9.99–15.23)	9.33 (7.34–11.31)	1.352 (0.927–1.972)	0.117

*Abbreviations*: *VA* Ventricular Arrhythmia

^a^Compared with secondary prevention patients

**Fig. 3:**
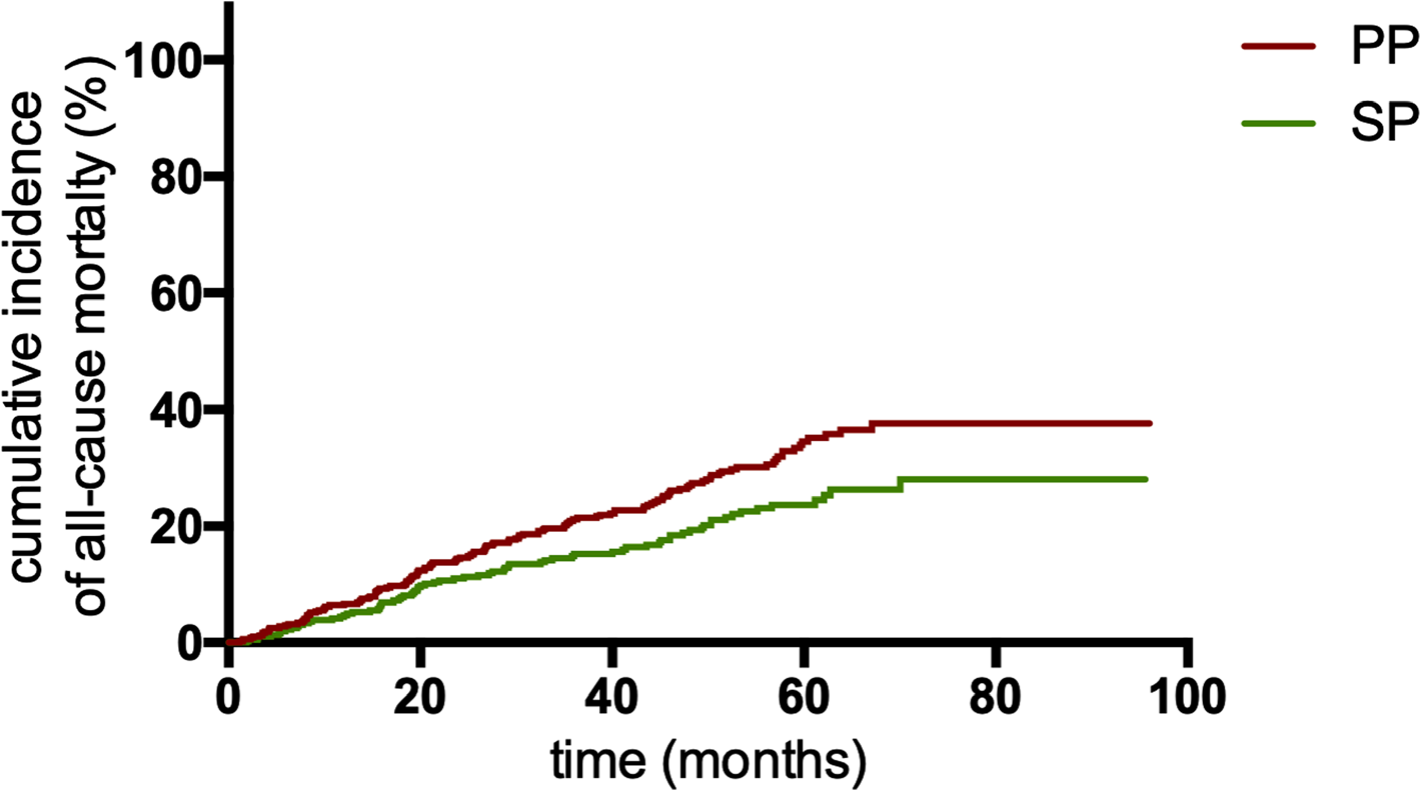
Kaplan-Meier estimates of the cumulative incidence of all-cause mortality between PP group and SP patients. Abbreviations: PP, primary prevention; SP, secondary prevention

**Fig. 4:**
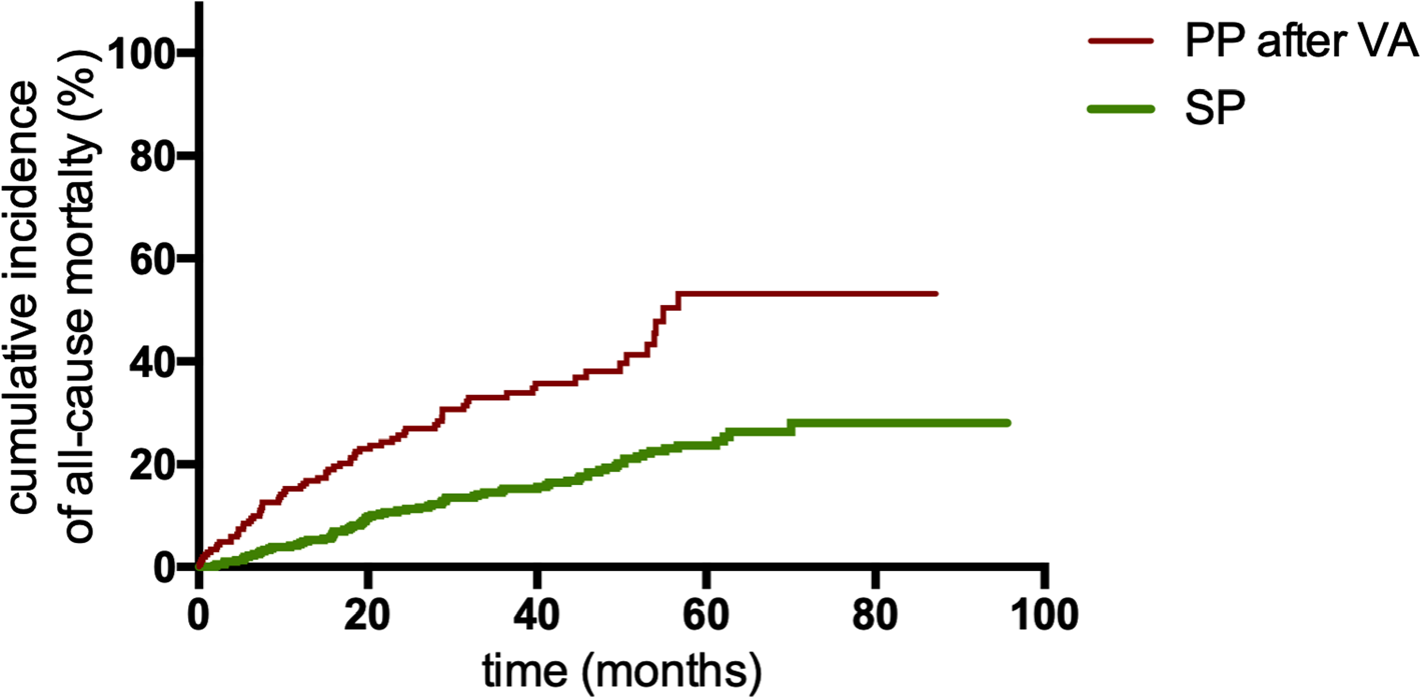
Kaplan-Meier estimates of the cumulative incidence of all-cause mortality between PP patients after appropriate device therapies and SP patients. Abbreviations: PP, primary prevention; SP, secondary prevention

Seventy-five (20.60%) patients died in the SP group, and 136 (28.87%) died in PP group. The cumulative incidence of all-cause mortality in the PP group was significantly higher than that in the SP group (*P* = 0.009, Fig. 3). Of 210 PP patients with first appropriate ICD therapy, 71 (33.81%) died after the first appropriate ICD therapy. The Kaplan-Meier analysis demonstrated that PP patients still had a higher cumulative mortality after the first appropriate ICD therapy than SP patients (*P*<0.001, Fig. 4). The rate of all-cause mortality in PP patients after the first appropriate ICD therapy was also significantly higher than that in SP patients (14.08 vs 5.40 deaths per 100 patient-years; IRR 2.607, 95% CI: 1.884–3.606; *P*<0.001; Table 3).

**Fig. 5:**
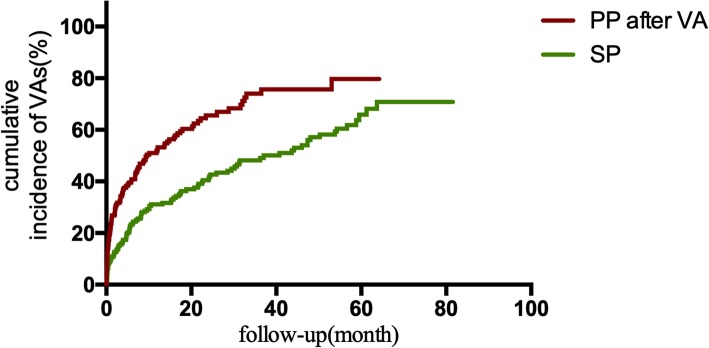
Kaplan-Meier estimates of the cumulative incidence of VAs between PP patients experienced appropriate device therapies and SP patients after propensity score matching. Abbreviations: VA, ventricular arrhythmia; PP, primary prevention; SP, secondary prevention

**Fig. 6:**
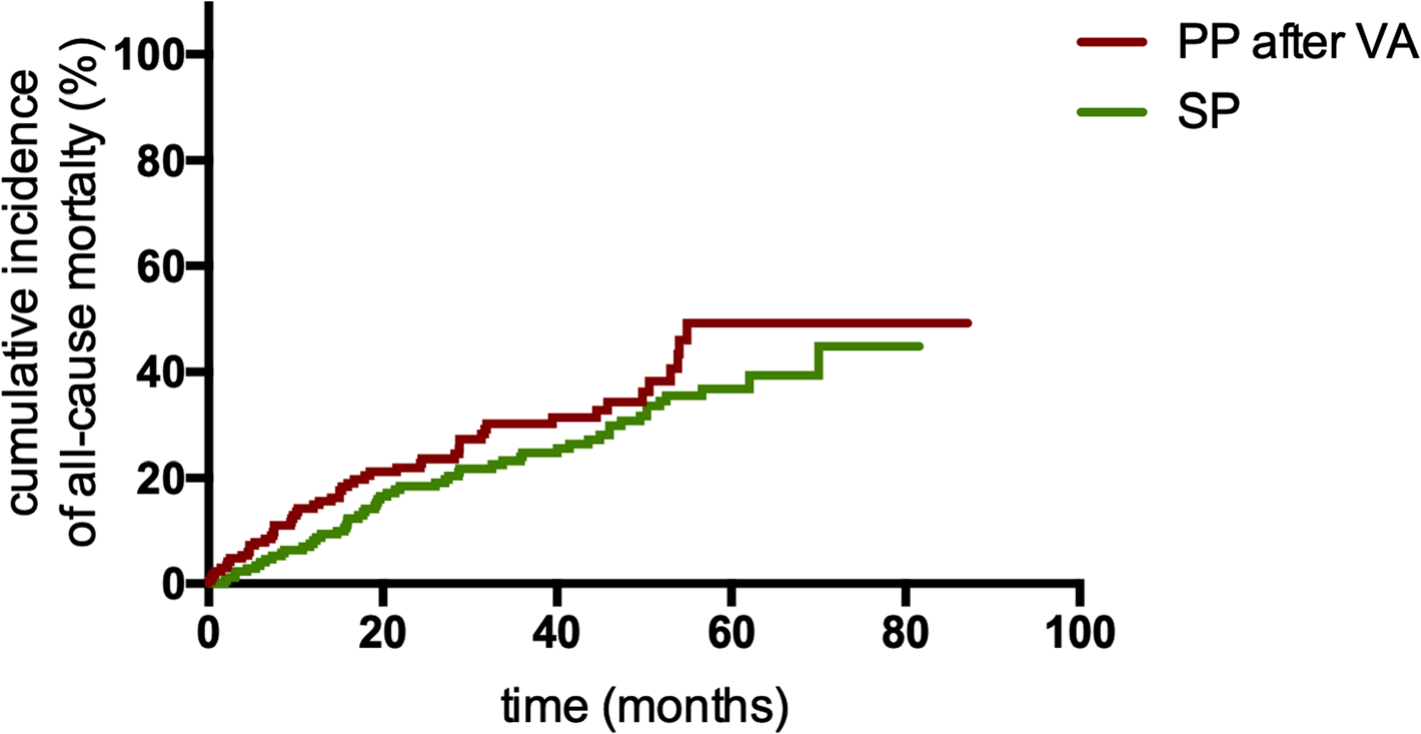
Kaplan-Meier estimates of the cumulative incidence of all-cause mortality between PP patients experienced appropriate device therapies and SP patients after propensity score matching. Abbreviations: PP, primary prevention; SP, secondary prevention

After propensity score matching, 173 PP patients experiencing VA and 173 SP patients with similar baseline characteristics were selected (Table 2). The rate of appropriate device therapies in PP patients after the first appropriate ICD therapy was still significantly higher than that in SP patients (41.80 vs 19.10 patients per 100 patient-years; IRR 2.491, 95% CI: 1.889–3.287; *P*<0.001; Table 3). Figure 5 shows the event-free survival between the two matched groups (*P*<0.001). However, the two groups had a similar rate of all-cause mortality (12.61 vs 9.33 deaths per 100 patient-years; IRR 1.352, 95% CI: 0.927–1.972; *P* = 0.117; Table 3). Figure 6 shows the survival curve between these two groups (*P* = 0.145).

## Discussion

The present study has two main findings. First, although the SP patients have a higher incidence of appropriate ICD therapy than the entire PP group, the PP patients are more prone to VA recurrence than SP patients. Second, the PP patients have a higher risk of all-cause mortality than the SP patients, but this difference diminished after adjusting the baseline characteristics.

Previous studies showed a higher occurrence of VA, requiring appropriate ICD therapy, in SP ICD patients than in PP patients. A study of 2471 ICD recipients demonstrated a cumulative 5-year incidence for appropriate therapy of 37 and 51% for PP and SP patients, respectively [6]. Compared with the PP group, the SP group was associated with a 74% increased risk of appropriate ICD therapy (*P*<0.001) [6]. An analysis of seven major ICD trials reported that appropriate device therapy rate ranged 54–64% during the follow-up periods of 36–45 months in SP studies, whereas a lower rate ranging 17–31% was observed during the follow-up periods of 24–29 months in PP studies [7]. These results were similar to the observed incidences in our study.

To the best of our knowledge, no prior studies compared the risk of subsequent VA in PP and SP patients. The PP patients after first appropriate ICD therapy was associated with a 2.88-fold incidence of subsequent appropriate episode. After adjusting the baseline difference, the higher incidence of VA in PP patients with appropriate device therapy still exists. There are two possible reasons of higher arrhythmia recurrence in PP patients after an ICD therapy. First, due to the prophylactic use of ICD, many PP patients who would have died because of malignant VA survived. Most SP patients survived probably due to relatively slow-rate VAs, whereas rapid-rate VAs will lead to SCD. PP patients with appropriate ICD therapy may have the highest risk of VAs in ICD recipients as a result of higher frequency and rapid rate of VAs than SP patients. Wilkoff et al. showed that PP patients had faster cycle lengths of appropriate ICD therapies than SP patients (303 ± 54 ms vs 366 ± 71 ms, *P* < 0.0001), wherein the LVEF was similar among the two groups [8]. Therefore, patients receiving ICDs for PP had a different clinical arrhythmia course than patients experiencing spontaneous VA. Second, ICD implantation solely based on LVEF included many patients who did not have high risk of SCD. Many PP patients did not experience appropriate ICD therapy until devices’ battery depletion or death. These patients decreased the incidence of VA in the entire PP population. Thus, PP patients after ICD therapy may have a higher risk due to previous VA episodes. This also demonstrated that the necessity of selecting “truly” high-risk patients. Currently, LVEF has been shown to be an inadequate tool to estimate the risk of SCD. LVEF could not fully reveal underlying substrate for arrhythmia. Previous studies have demonstrated that LVEF is not consistent with myocardial fibrosis detected by cardiac magnetic resonance [9] and the electrical instability detected by electrophysiological study [10]. This suggests that LVEF combined with other indicators may help more accurately screen high-risk patients.

The all-cause mortality rate of PP patients was not necessarily lower than that in SP patients in prior studies. In an ICD registry of 7020 patients, the incidence of all-cause mortality was comparable for both indications (6.87 per 100-person years in the PP group vs. 7.31 per 100-person years in the SP group, *P* = 0.178) [11]. Van Welsenes reported a higher risk of all-cause mortality in PP patients than in SP patients over 5 years of follow-up (HR: 1.2, 95% CI: 1.0–1.5, *P* = 0.05) [6]. In our study, PP ICD recipients after appropriate device therapies exhibited a higher risk of experiencing life-threatening arrhythmic events requiring appropriate ICD therapy than SP patients. The negative impact of appropriate ICD therapy on mortality has been widely recognized [11–13]. Therefore, the heavier burden of appropriate episodes contributes to the higher mortality. In contrast, considering that PP patients had more advanced heart failure at baseline, higher mortality rates for PP patients were expected. Severe left ventricular dysfunction is related to an increased risk of non-arrhythmic death. Furthermore, after adjusting the baseline difference, PP ICD recipients after appropriate device therapies had a similar all-cause mortality rate to SP patients. The incidence of all-cause mortality increased more after propensity score matching (5.40 deaths per 100 patient-years vs 9.33 deaths per 100 patient-years). This may be due to the decline in LVEF after matching.

Our study revealed that once a PP patient underwent appropriate ICD therapy and was declared an SP prevention patient, this patient’s risk of subsequent appropriate ICD therapy and death increased. PP patients after appropriate ICD therapy should receive more attention. Current treatment strategies to prevent VT in ICD patients mainly include antiarrhythmic drugs and catheter ablation, but the optimal suppressive therapy remains to be determined. In ICD recipients, compared with solely standard medical therapy, both amiodarone and ablation were efficacious in reducing recurrent VT [14]. However, the reduced risk of VT did not lead to a survival benefit, with a potential for increased mortality with amiodarone [14]. Ablation can be effective, but patient selection and VT recurrence requiring repeat ablation should be considered. Early ablation may be appropriate in some clinical situations, such as patients presenting with relatively slow VT below ICD detection rate, electrical storms, or hemodynamically stable VT or very selected patients with left ventricular assist devices [15].

Several limitations need to be considered. The cause of death is unadjudicated and confirmed by family members. The exact classification of death may be not reliable in the present study, particularly for SCD. Second, the majority of participants in this study did not receive cardiac magnetic resonance and electrophysiological study, which might help clarify the differences of substrate for arrhythmia in both indications. Third, some ICD programming settings might be not optimal in current view. For instance, a detection interval of 12/16 in VF zone and the slowest tachycardia therapy zone limit (above 170 bpm) in PP patients may lead to a high rate of ICD therapy [16, 17]. Finally, the patients’ clinical characteristics may be different from baseline when they underwent first device therapy during the follow-up, and our study did not collect data, such as LVEF and medication modifications, after the first ICD therapy. However, in most survival analysis, the baseline characteristics were not considered to be time-varying during the follow-up. The Kaplan-Meier curves were also calculated between patients after appropriate ICD therapy and patients who never experienced ICD therapy in previous studies [18, 19]. Because this is a retrospective study, not specifically designed to assess endpoints reported within this manuscript, prospective studies are required to further confirm the results of the study.

## Conclusions

Once PP patients undergo appropriate ICD therapy, they will be more prone to VA recurrence and death than SP patients.

## Data Availability

The datasets generated and analysed during the current study are not publicly available due to the Fuwai Hospital regulations, but are available from the corresponding author on reasonable request.

## Abbreviations

CRT-D: Cardiac resynchronization therapy defibrillator
HM: Home monitoring
ICD: Implantable cardioverter defibrillator
IRR: Incidence rate ratio
LVEF: Left ventricular ejection fraction
SCD: Sudden cardiac death
VA: Ventricular arrhythmia
VT: Ventricular tachycardia
